# Supercapacitor Performance of Nickel-Cobalt Sulfide Nanotubes Decorated Using Ni Co-Layered Double Hydroxide Nanosheets Grown in Situ on Ni Foam

**DOI:** 10.3390/nano10030584

**Published:** 2020-03-23

**Authors:** Chen Xin, Li Ang, Farayi Musharavati, Fadi Jaber, Li Hui, Erfan Zalnezhad, Sungchul Bae, Kwan San Hui, Kwun Nam Hui

**Affiliations:** 1 Department of Mechanical Convergence Engineering, Hanyang University, 222 Wangsimni-ro, Seongdong-gu, Seoul 04763, Korea; 2014125024@hanyang.ac.kr (C.X.); 2016109862@hanyang.ac.kr (L.A.); 2 Department of Mechanical and Industrial Engineering, College of Engineering, Qatar University, Doha 2713, Qatar; farayi@qu.edu.qa; 3 Department of Biomedical Engineering, Ajman University, Ajman 346, UAE; f.jaber@ajman.ac.ae; 4 Department of Chemical Engineering, Hanyang University, 222 Wangsimni-ro, Seongdong-gu, Seoul 04763, Korea; l251717756@hmail.hanyang.ac.kr; 5 Department of Biomedical Engineering, University of Texas at San Antonio, TX 78249, USA; 6 Department of Architectural Engineering, Hanyang University, Seoul 04763, Korea; 7 Engineering, Faculty of Science, University of East Anglia, Norwich, NR4 7TJ, UK; 8 Joint Key Laboratory of the Ministry of Education, Institute of Applied Physics and Materials Engineering, University of Macau, Avenida da Universidade, Taipa 999078, Macau, China

**Keywords:** NCS@NCOH nanotubes, asymmetric supercapacitor, positive electrode material

## Abstract

In this study, to fabricate a non-binder electrode, we grew nickel–cobalt sulfide (NCS) nanotubes (NTs) on a Ni foam substrate using a hydrothermal method through a two-step approach, namely in situ growth and an anion-exchange reaction. This was followed by the electrodeposition of double-layered nickel-cobalt hydroxide (NCOH) over a nanotube-coated substrate to fabricate NCOH core-shell nanotubes. The final product is called NCS@NCOH herein. Structural and morphological analyses of the synthesized electrode materials were conducted via SEM and XRD. Different electrodeposition times were selected, including 10, 20, 40, and 80 s. The results indicate that the NCSNTs electrodeposited with NCOH nanosheets for 40 s have the highest specific capacitance (SC), cycling stability (2105 Fg^−1^ at a current density of 2 Ag^−1^), and capacitance retention (65.1% after 3,000 cycles), in comparison with those electrodeposited for 10, 20, and 80 s. Furthermore, for practical applications, a device with negative and positive electrodes made of active carbon and NCS@NCOH was fabricated, achieving a high-energy density of 23.73 Whkg^−1^ at a power density of 400 Wkg^−1^.

## 1. Introduction

With the continued global increase in population and the need for sustainable socio-economic development to fulfill the high energy demand, energy production, conversion, and storage have become areas of focus [1]. The energy used in current day-to-day activities is still highly dependent on fossil fuels, which are mainly used for transportation, heating, cooling, and power. This can lead to a sharp rise in energy costs and a continual increase in the prices of crude oil and natural gas [2]. In addition, the use of energy from such sources will create irreversible environmental problems. With a growing focus on carbon emissions and clean energy, the provisioning of reliable energy supplies without the production of excessive carbon has become a key requirement [3]. Supercapacitors have the characteristics of traditional capacitors and secondary batteries [4]. Nanostructured metal sulfides, which offer significant advantages in various energy-storage devices, are attracting considerable interest from researchers [5]. NiCoS is a promising candidate owing to its excellent energy storage performance, nontoxicity, and use of low-cost Co and Ni ions [6]. Binary metal sulfides have a narrower band gap and higher electrical conductivity than metal oxides, indicating a better electrochemical performance [7]. In addition, the severe aggregation of NiCoS can decrease the rate of utilization of electrode materials, which is unfavorable to electron transfer and ion diffusion [8]. Hence, NiCoS-type electrode materials with various structures, such as nanosheets [9], nanoparticles [10], and nanotubes [11], have been widely investigated to solve the challenges mentioned earlier. Qiu et al. [12] prepared nickel–cobalt sulfide (NCS) using one- and two-step hydrothermal methods. The obtained pseudosupercapacitor electrodes deliver a higher specific capacitance of 1492 Fg^−1^ at 1 Ag^−1^. LDH, which offers excellent anion exchange performance and thermochemical properties, has attracted interest globally and has already found use in numerous fields, including ion exchange [13], water treatment [14], composites [15], catalytic materials [16], active molecule storage [17,18], and the preparation of chemical topology [19]. For example, in carbon materials [20], metal oxides [21], and conductive polymers [22], each material exhibits different advantages and disadvantages; hence, to increase the performance and electrochemical properties of the electrodes, a combination of materials is necessary [23].

Herein, a new core-shell nanostructure NCS@NCOH electrode was prepared using a simple two-step process. A rational core-shell nanostructure was also designed by adjusting the electrodeposition time of the NCOH to enhance the ratio of ions in the electrode materials and the electron transfer. A nanostructure (core-shell) can quickly transport electrons and proliferate ions of a highly specific capacity and large surface area on NCOH nanosheets. An asymmetric supercapacitor was fabricated using an NCS@NCOH nanotube as a positive electrode and a carbon nanotube as a negative electrode. Through various experiments, it was concluded that an electrodeposited double-layered hydroxide nanotube on NCS nanowires shows good potential as a supercapacitor electrode material. 

## 2. Experimental Details

### 2.1. Chemicals

The chemical materials, which were of systematic grade, were utilized as received. The chemicals cobalt nitrate hexahydrate (Co(NO_3_)_2_·6H_2_O), nickel chloride hexahydrate (NiCl_2_ · 6H_2_O), urea (NH_2_CONH_2_), sodium sulfide nonahydrate (Na_2_S·9H_2_O), nickel nitrate hexahydrate (Ni(NO_3_)_2_ · 6H_2_O), HCl, and cobalt chloride hexahydrate (CoCl_2_ · 6H_2_O) were provided by Sigma Aldrich (Seoul, South Korea), and nickel foam was purchased from Jiayisheng Co. (Hong Kong, China).

### 2.2. Fabrication of NCS Nanotubes on the Substrate

The substrate (Ni foam) was cut into 1 × 1 cm^2^ samples and cleaned in 3 M HCl, ethanol, and double distilled water to remove any oxides and impurities and then dried in an oven overnight at 70 °C. NCS nanotubes were fabricated using a hydrothermal technique in two steps. In the first step, 12 mmol urea, 4 mmol cobalt chloride hexahydrate, 2 mmol nickel chloride hexahydrate, and 35 mL of distilled water were mixed using a stirrer. The blended solutions were poured into a 75 mL Teflon autoclave with the nickel foam substrates residing inside and then transferred to an oven and maintained at 110 °C for 8 h. Finally, the specimens were rinsed with distilled water and ethanol and then kept in the oven overnight at 70 °C. In the second step, the samples obtained from the first step of the hydrothermal treatment were placed in a 75 mL Teflon autoclave, and 35 mL of a 0.2 M Na_2_S solution was kept in the oven at 110 °C for 15 h. The final products were rinsed with ethanol and distilled water to remove any residue and then kept in the oven overnight to obtain the NCS nanotubes.

### 2.3. Fabrication of NCS@NCOH Core-Shell Nanotube Arrays

An electrodeposition method was used to decorate NCSNTs with an NCOH core-shell. A nickel-cobalt sulfide nanotube sample, calomel electrode, and platinum foil were utilized as the working, reference, and counter electrodes, respectively. The NCOH nanosheets were electrodeposited at a constant potential of −1.2 V for different times (10–80 s). The electrodeposition electrolyte was provided by mixing 2 mmol of nickel nitrate hexahydrate and 4 mmol of cobalt nitrate hexahydrate in 50 mL of distilled water. The electrodeposited sample (electrode) was washed and cleaned with double distilled water and ethanol a few times and kept in a vacuum oven at 70 °C for 12 h. 

### 2.4. NCS@NCOH//AC Asymmetric Supercapacitor (ASC) Device Fabrication

The NCS@NCOH//AC ASCs were created using NCS@NCOH NTAs and carbon nanotubes as positive and negative electrodes. A potassium hydroxide (6 M) solution was chosen for the electrolyte. Acetylene black, polyvinylidene fluoride (PVDF), and activated carbon (AC) were used to create a negative electrode mixture with a mass ratio of 1:1:8, which was then applied evenly to a nickel foam sample. The negative electrode was kept in a vacuum oven at 60 °C for 12 h.

Before assembling and installing the asymmetric supercapacitors, the masses of the negative and positive electrodes were well-adjusted and balanced according to Equation (1) [24]:

(1) $$\frac{m_{+}}{m_{-}}=\frac{C_{s}-\Delta V_{-}}{C_{s}+\Delta V_{+}},$$

where Δ*V*, *C_s_*, and *m* are the voltage range for the negative and positive electrodes, the specific capacitance of a single electrode at a scan rate of 2 A g^−1^, and the mass, respectively. The mass burden of the NCS@NCOH//AC ASCs was approximately 9.68 mg.

### 2.5. Material Characterization

SEM (with an applied voltage of 5 kV) and XRD were used to analyze the structure and morphology of a working electrode. An XRD analysis was conducted on a Bruker D8 Advance XRD Aldrich (Seoul, South Korea), utilizing Cu Kα radiation with an incident X-ray beam with a wavelength of 0.154056 nm at 30 mA and 40 kV. The scanning steps and speed were 0.02° and 5°/min^−1^, respectively.

### 2.6. Electrochemical Experiments

The electrochemical experiments were implemented through a three-electrode system in which the NCS@NCOH NTAs specimen, Hg/HgO, and platinum foil were the working, reference, and counter electrodes, respectively. For the electrochemical experiments, a 3 M KOH solution was used. A ZIVE SP2 electrochemical workstation (10 μHz to 4 MHz) was applied to evaluate the electrochemical performance of the samples using cyclic voltammetry (CV), galvanostatic charge-discharge (GCD), and electrochemical impedance spectroscopy (EIS). The voltage windows for the negative activated carbon electrode and the positive NCS@NCOH electrode were −1.0–0 and 0–0.6 V, respectively. 

Equations (2) and (3) were used to obtain the capacitance (from the GCD curves).

For the electrodes,

(2) $$C_{s}=\frac{I\Delta t}{mV},$$

For the ASCs,

(3) $$C_{ASC}=\frac{I\Delta t}{MV},$$

Here, *C_s_* is the specific capacitance, *C_ASC_* is the ASC capacitance, *I* is the discharge current, Δ*t* is the discharge time, and *V* is the change in voltage. Similarly, m and M are the mass of the electroactive material on the single electrode and the total mass of the electroactive materials based on both the negative and positive electrodes (*M* = *m*_+_ + *m*_−_).

Equations (4) and (5) were used to calculate the power density and energy (based on the total mass of the two electrodes in the ASCs):

(4) $$E=\frac{C_{ASC}V^{2}}{2},$$

(5) $$p=\frac{E}{\Delta t}=\frac{C_{ASC\;}V^{2}}{2\Delta t},$$

where Δ*t*, *P*, *C_ASC_*, *E*, and *V* are the discharge time, power density, ASC capacitance, energy density, and voltage change, respectively.

## 3. Results and Discussion

### 3.1. Sample Characterizations

**Figure 1:**
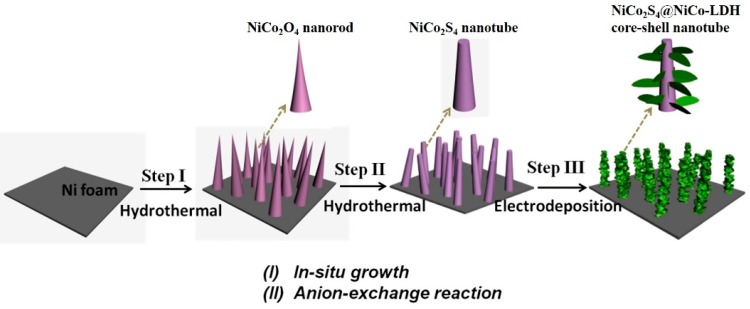
Schematic illustration of formation of NCS@NCOH NTAs on Ni foam.

Figure 1 presents a schematic of the experimental procedure used for the fabrication of an NCS@NCOH core-shell nanotube on Ni foam. First, NCS nanotubes are created on the Ni foam through a hydrothermal procedure in two steps, namely in situ growth of a NiCo_2_O_4_ nanoneedle and an anion-exchange reaction of the NCS nanotube. Second, the NCS nanotube coated Ni foam was used to deposit NCOH nanosheets through electrodeposition.

**Figure 2:**
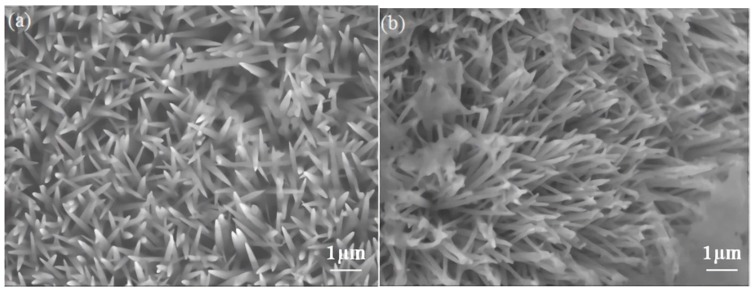
(**a**) FESEM image of NCO nanorod arrays and (**b**) FESEM image of NCS nanotube arrays on Ni foam.

Figure 2a presents an FESEM image of NCO with a nanoneedle morphology homogeneously distributed over the Ni foam substrate. As Figure 2 indicates, in an anion-exchange reaction, owing to their porous structure, the nanoneedle arrays serve as an outward diffusion pathway for nickel-cobalt oxide. Furthermore, during a hydrothermal reaction (after reacting with Na_2_S), it can clearly be observed that the sharp ends of the nanoneedles disappear (Figure 2b). 

**Figure 3:**
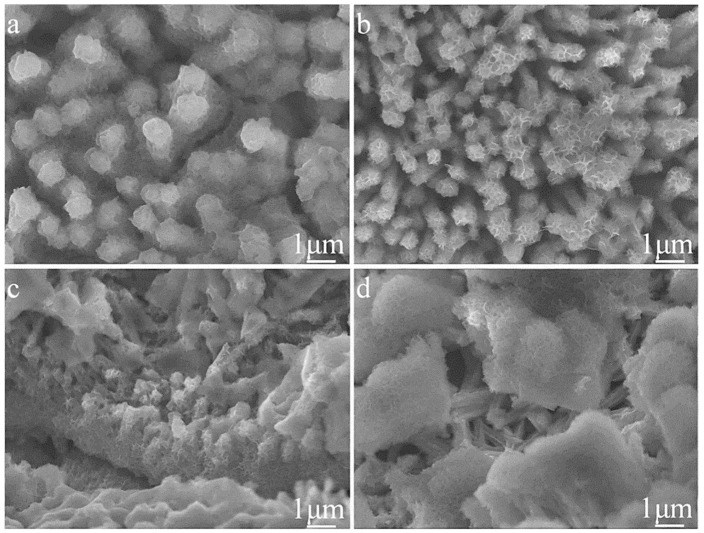
FESEM images of NCS@NCOH_n core-shell nanotube arrays: (**a**) *n* = 1, (**b**) *n* = 2, (**c**) *n* = 3, and (**d**) *n* = 4.

To achieve a better understanding and closer analysis of the morphologies and structures of the NCS@NCOH core-shell nanotubes, electrodeposition was conducted for different times, including 10, 20, 40, and 80 s, while maintaining a constant temperature and solution concentration. Figure 3 presents an FESEM image of NCS nanotubes composed of thin NCOH nanosheets developed using different electrodeposition times. As illustrated in Figure 3a, although the NCOH nanosheets have been inspected, the core-shell construction is not very clear. This results in a small amount of NCOH with an electrodeposition time of only 20 s. With an increase in the deposition time, more NCOH is involved. As illustrated in Figure 3c, after 60 s of electrodeposition, the NCS nanotubes are coated with a large number of NCOH nanosheets, resulting in a smaller vacancy amid the core-shell nanotubes. Moreover, as presented in Figure 3d, after 80 s of electrodeposition, the pores between nanotubes are filled. The most optimal morphologies after a 40 s electrochemical deposition time, shown in Figure 3b, achieve a better electrochemical performance, which will be discussed later. The NCOH nanosheets are interrelated, thereby creating a highly porous structure. At a large scale, well arranged channels are generated by the vacancy amid each NCOH nanosheet decorated nanotube. The open space between the neighboring nanoflakes and the neighboring NCS@NCOH core-shell nanostructures allows an easy approach of ions to the electrolyte/electrode port and makes it possible for Faradic reactions to occur during the energy conversion process.

**Figure 4:**
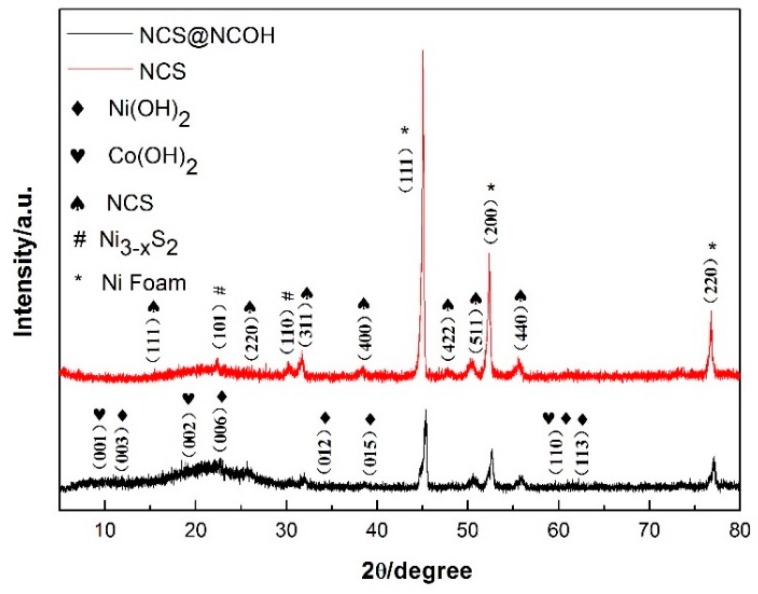
XRD patterns of the NCS nanotube arrays and NCS@NCOH core-shell nanotube arrays on Ni foam.

Another observation was conducted to examine the structure of the working electrode through an XRD analysis. Figure 4 presents the XRD patterns of the NCS and NCS@NCOH core-shell nanotube arrays coated onto the nickel foam. Peaks at (111), (200), and (220) were generated from the nickel foam substrate at 44.7°, 52.1°, and 76.5°, respectively. In addition, the diffraction peaks at 16.3°, 26.7°, 31.6°, 38.3°, 47.4°, 50.5°, and 55.3° correspond respectively to the (111), (220), (311), (400), (422), (511), and (440) planes of the NCS (the cubic-type) phase (JCPDS Card # 20-0782). Moreover, the weak peaks at 9.2°, 19.2°, and 58.2° coincide with the (001), (002), and (110) planes of cobalt(II) hydroxide (JCPDS card #51-1731). Similarly, the weak peaks at 11.4°, 22.7°, 34.4°, 38.8°, 60.0°, and 61.3° coincide with the (003), (006), (012), (015), (110), and (113) planes of the Nickel(II) hydroxide, respectively (JCPDS card #38-0715). The peaks corresponding to the two spectrums are superimposed to form NCOH peaks, as illustrated in Figure 4. Moreover, other studies report that the diffraction peaks at 21.5° and 30.2° match the (101) and (110) planes of Ni_3-x_S_2_ (JCPDS card #14-0358) [25]. 

**Figure 5:**
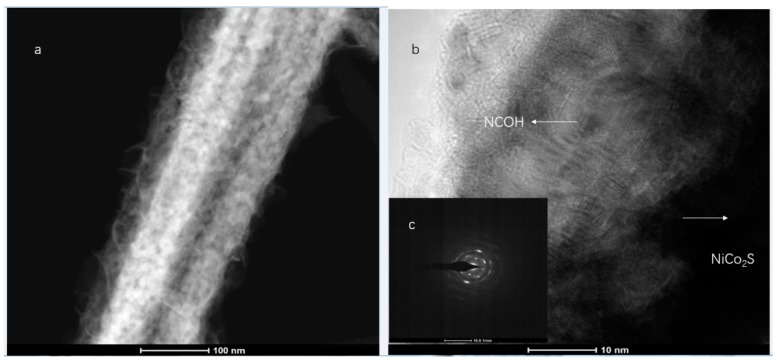
(**a**) TEM and (**b**) HRTEM images of individual NiCo2O4@NiCo2O4 hierarchical nanostructures and (**c**) inset showing the SAED.

**Figure 6:**
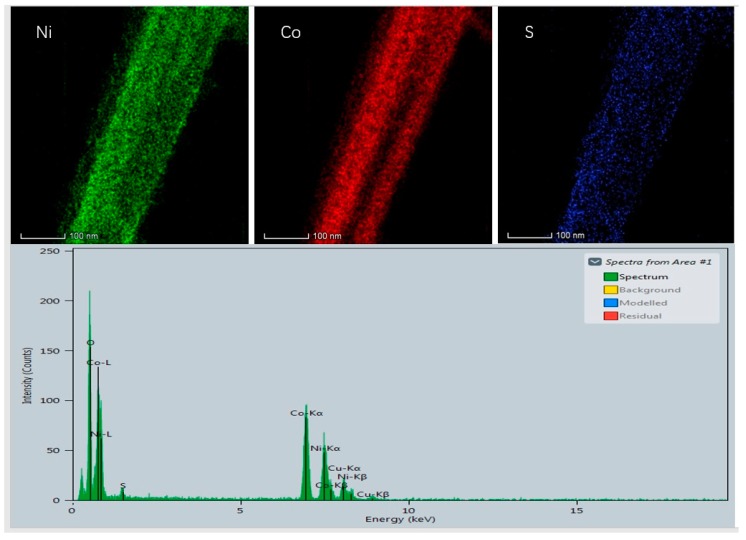
Corresponding EDS mapping results.

For a further study of the morphology and microstructure of the as-synthesized NCS@NCOH samples, a transmission electron microscopy (TEM) analysis was conducted under different magnifications. As illustrated in Figure 5, the NCS was clearly wrapped in NCOH nanotubes, and a core-shell nanotube structure can clearly be observed. High-resolution transmission electron microscopy (HRTEM) and selected area electron diffraction (SAED) images are presented in Fig. 5 and its inset, respectively. The SAED pattern (inset) is also consistent with the XRD results, and the diffraction rings reveal the growth of NCOH on the surface of the NCS. In addition, the core–shell structure of the NCS@NCOH nanowires was further identified through an EDS mapping analysis of Co, Ni, O, and S in the STEM (Figure 6).

### 3.2. Electrochemical Characterizations of the Working Electrode

**Figure 7:**
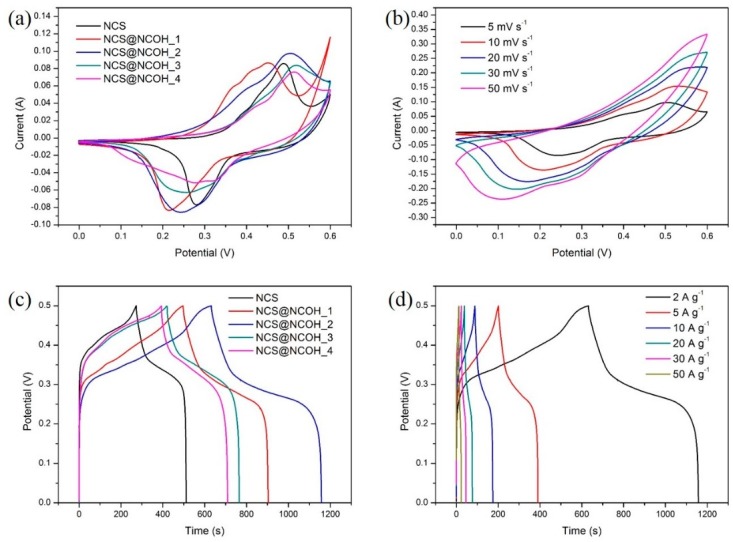
(**a**) CV curves of the NCS nanotube arrays and NCS@NCOH core-shell nanotube arrays with different electrodeposition time at 5 mV s^−1^, (**b**) CV curves of NCS@NCOH_2 core-shell nanotube arrays at various scan rates, (**c**) GCD curves of NCS nanotube arrays and NCS@NCOH core-shell nanotube arrays with different electrodeposition times at 2 A g^−1^, and (**d**) GCD curves of NCS@NCOH_2 core-shell nanotube arrays at different current densities.

The electrochemical performance (battery-type electrodes, energy storage) of Ni foam coated with NCS@NCOH core-shell nanotubes was examined using GCD and CV methods in a conventional three-electrode system. Figure 7a shows the CV curves of the NCS nanotube and NCS@NCOH core-shell nanotube coatings on the Ni foam for different samples with different electrochemical deposition times of 10, 20, 40, and 80 s, which are labeled as NCS@NCOH _1, NCS@NCOH _2, NCS@NCOH _3, and NCS@NCOH _4, respectively. The scanning range, compared to HgO, differs from 0 to 0.6 V. It is clear that at various electrodeposition times, the integral areas of the NCS@NCOH’ cyclic voltammetry curves are dissimilar and the trends of the cyclic voltammetry curves are similar. In Figure 7a, the NCS@NCOH_2 sample exhibits excellent charge transfer compared to the other samples. The enhancement in the specific capacitance and rate capability of the NCS@NCOH _2 sample mainly originates from the hierarchical porous architecture, which enables a sufficient exposure of the pseudocapacitance-active components. Furthermore, the chemical binding between the NCS core and the NCOH shell facilitates the charge transport process between the active components and the current collector, which contributes to the superior rate capability. The change in potential (∆*E*_a,c_) amid the anodic and cathodic peaks of the electrode was used to calculate the electrochemical redox reaction reversibility (that is, the smaller the value of ∆*E*_a,c_, the greater the reversibility). Even with a tighter potential window, nickel-cobalt sulfide (NCS) nanotubes (NCSNTs) decorated with NCOH nanosheets demonstrate a greater imploded cyclic voltammetry area compared to NCSNTs alone, which can be attributed to the complementary capacity from the nickel-cobalt layered double hydroxide layer (Co_x_Ni_(1−x)_(OH)_2_ + OH^−^ ↔ Co_x_Ni_(1−x)_OOH + H_2_O + e^−^) [26]. It should be noted that variations in specimens may originate from the alterations in the trends of electrode polarization. Figure 7b presents the CV curves of the NCS@NCOH_2 core-shell nanotube specimen with a 0–0.6 V potential window (compared to HgO) at various rates of 5 to 50 mV s^−1^. Moreover, the redox peaks presented in the cyclic voltammetry curves under various scan rates indicate the electrochemical behavior of the sulfide electrodes. The structure of the redox peaks is perhaps attributed to the Ni^2+^/Ni^3+^ and Co^2+^/Co^3+^/Co^4+^ Faradaic processes (reactions (6), (7), and (8)) [27]:

(6) $$CoS+OH^{-}↔CoSOH+e^{-},$$

(7) $$CoSOH+OH^{-}↔CoSO+H_{2}O+e^{-},$$

(8) $$NiS+OH^{-}↔NiSOH+e^{-},$$

The GCD increases slowly with an enhancement in the scan rate. The anodic and cathodic peaks divert toward the positive and negative potentials, respectively, which is an evidence supporting that quick redox reactions occur at the interface of the electroactive material/electrolyte. Figure 7c presents a comparison of the GCD curves of the nickel-cobalt sulfide nanotubes and NCSNTs decorated with a NCOH core-shell at different electrodeposition times at 2 Ag^−1^ and with a potential window of 0–0.5 V. As illustrated in Figure 7c, the NCS@NCOH_2 electrode displays a more stable discharge plateau than the other samples, leading to an extended discharge time and thus a higher SC. Following Equation (2), the SC values of NCS, NCS@NCOH_1, NCS@NCOH_2, NCS@NCOH_3, and NCS@NCOH_4 electrodes are calculated to be 953.4, 1624.4, 2105.1, 1376.9, and 1261.8 Fg^−1^, respectively. Figure 7d shows the charge-discharge curves and SC of NCS@NCOH_2 core-shell nanotube arrays at various CDs. As calculated, the values of SC of the NCS@NCOH_2 electrode are 2105.1, 1887.7, 1708.3, 1503.5, 1364.7, and 1128.3 Fg^−1^ at 2, 5, 10, 20, 30, and 50 Ag^−1^ current densities, respectively. Once the CD increases, the electrochemical reaction is noticeably slower than the rate of electron transfer, and thus, the electrode materials do not have sufficient time to contribute to a redox reaction, resulting in a reduction in capacity [28]. 

**Figure 8:**
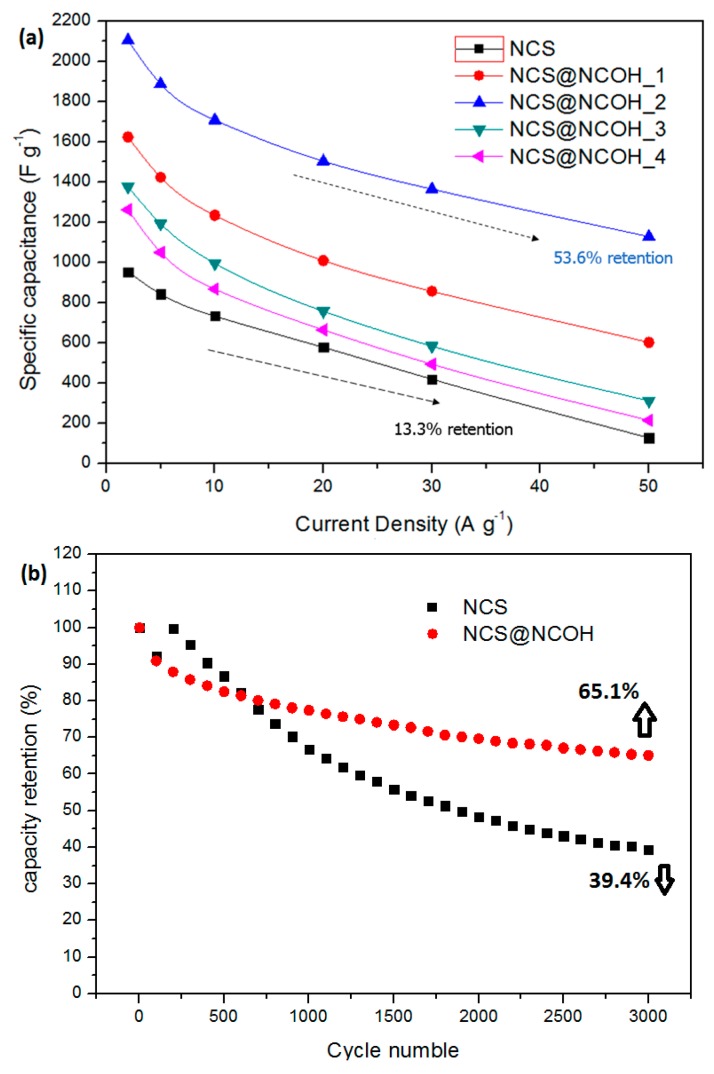
(**a**) Specific capacitance of NCS@NCOH core-shell nanotube array electrodes with different electrodeposition times and (**b**) cycling performance of NCS and NCS@NCOH at 50 A g^−1^ for 3,000 cycles.

Figure 8a shows that the NCSNTs decorated with NCOH nanosheets electrodeposited as an electrode for 40 s achieve a high SC of 2105.1 Fg^−1^ at 2 Ag^−1^. As can be seen, when enhancing the CD from 2 to 50 Ag^−1^, the SC decreases to 1128.3 Fg^−1^, corresponding to a 53.6% capacity retention. Regarding the NCSNTs electrode, the SC is 953 F g^−1^ at 2 A g^−1^ and drops to 127 F g^−1^ at 50 Ag^−1^. In comparison, an NCSNT-decorated NCOH nanosheet electrode is considerably better than a nickel-cobalt sulfide nanotube electrode with only a 13.3% retention. The cycling stabilities of the NCS@NCOH and NCS electrodes were tested at 50 Ag^−1^ CD for over 3000 successive cycles, and Figure 8b demonstrates the number of cycles versus the capacitance retention. In the initial 500 cycles, the capacitances of both electrodes exhibit clear deteriorations. Subsequently, the capacitance-decay tendency of the NCS@NCOH electrode weakens. However, the capacitance of the NCS electrode still maintains a relatively high attenuation trend that is not weakened until >2000 cycles. The capacitance retention of the NCS@NCOH electrode is 65.1% after 3000 cycles. This is also significantly better than the pure NCS electrode with only 39.4% retention (after 3000 cycles). NCS@NCOH demonstrates the best performances (capacitive) and significant cycling stability. Furthermore, the SCs of the NCS@NCOH electrode obtained in this study are also better or comparable to the earlier reported NCS@NCOH electrodes (the SC was 1765 Fg^−1^ at 1 Ag^−1^, and after 2000 cycles, the capacity retention was 56%) [29]. 

**Figure 9:**
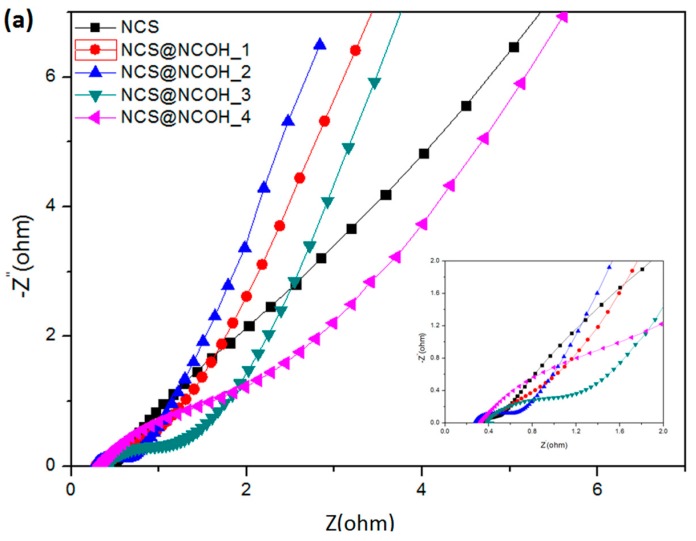

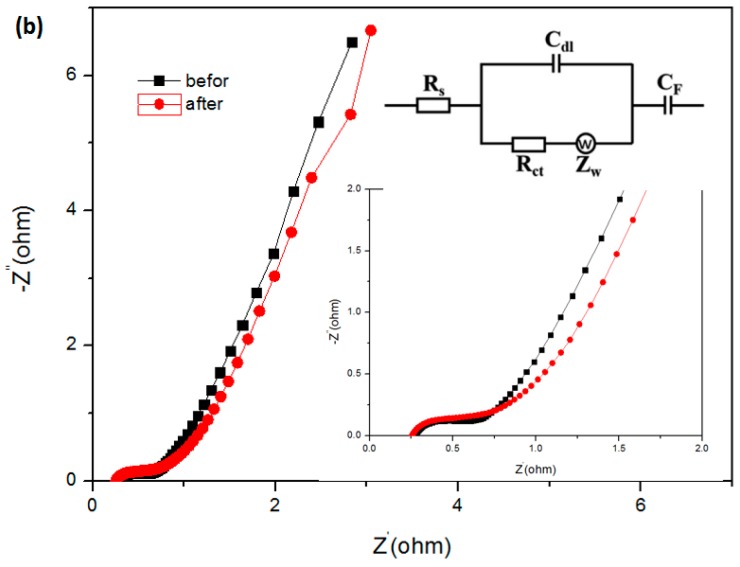
(**a**) Nyquist plots of NCS nanotube arrays and NCS@NCOH core-shell nanotube array electrodes with different electrodeposition times and (**b**) Nyquist plots of NCS@NCOH electrode before and after cycling tests.

The EIS tests were conducted with the frequency range of 0.01 Hz to 100 kHz to appraise the performance of the ion transport of the working electrode materials for supercapacitor application. Figure 9 presents the proposed equivalent circuit for the measured impedance data that involve the internal resistance (*R_s_*), double-layer capacitance (*C_dl_*), Faradic charge transfer resistance (*R_ct_*), Warburg diffusion element (*Z_w_*), and pseudocapacitance (*C_F_*). Equations (9) and (10) show the overall impedance, *Z*, of the equivalent circuit in Figure 9:

(9) $$Z=R_{s}+\frac{1}{j\omega C_{dl}+\frac{1}{R_{ct}+Z_{w}}}-j\frac{1}{\omega C_{F}},$$

(10) $$Z_{w}=\frac{W}{\sqrt{j\omega }},$$

where *j* is the imaginary unit, *u* is the angular frequency (Hz), and *W* is the Warburg parameter in units of Ωs^−1/2^. Here, *W* is an increasing function of resistance for electrolyte transport in a porous electrode. At sufficiently high frequencies, the overall impedance can be reduced to Equation (11), corresponding to a locus showing a semicircle that intercepts the real axis at *Rs* and *R_s_* + *R_ct_* in the Nyquist plot.

(11) $$Z=R_{s}+\frac{1}{j\omega C_{dl}+\frac{1}{R_{ct}}},$$

Figure 9a presents the Nyquist plots of versatile mixture electrodes. At a high frequency, the semicircle of the NCS@NCOH_2 electrode is smaller than that of the other samples, whereas at a low frequency, the NCS@NCOH_2 electrode has the largest slope, with *R_s_* and *R_ct_* values of 2.5 and 2.9 Ω, respectively, of an NCS@NCOH_2 electrode. These results indicate that double-layered hydroxide has a high electrochemical capacity owing to its fast and reversible redox reactions. The coating of NCOH on a nickel-cobalt sulfide (NCS) nanotube surface directly in contact with the electrolyte reduces the resistance, leading to a quick contact of the surface of the electrode materials with the ions of the electrolyte. As demonstrated in Figure 9b, the impedances of the NCS@NCOH electrodes before and after the cycling tests were measured to evaluate the performances of the electrodes. The semicircle is clearly larger after the cycling test than it is before the test. This indicates that during the test, the electrode material produces irreversible reactants leading to an increased resistance. It is clear that the curves have a semicircle transecting *R_C_* circuit that specifies the capacitance in parallel with the resistance. In a region with a lower frequency, ion penetration into the pores or surface of the electrode was achieved. The perpendicular line represents the control of the capacitive action.

### 3.3. Performance Evaluation of Asymmetry Supercapacitor Based on Active Carbon and NCS@NCOH 

**Figure 10:**
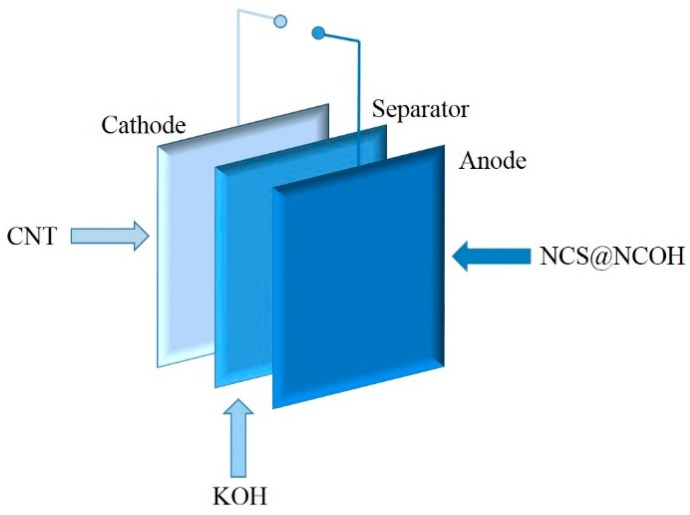
Schematic illustration of assembled asymmetric supercapacitors.

**Figure 11:**
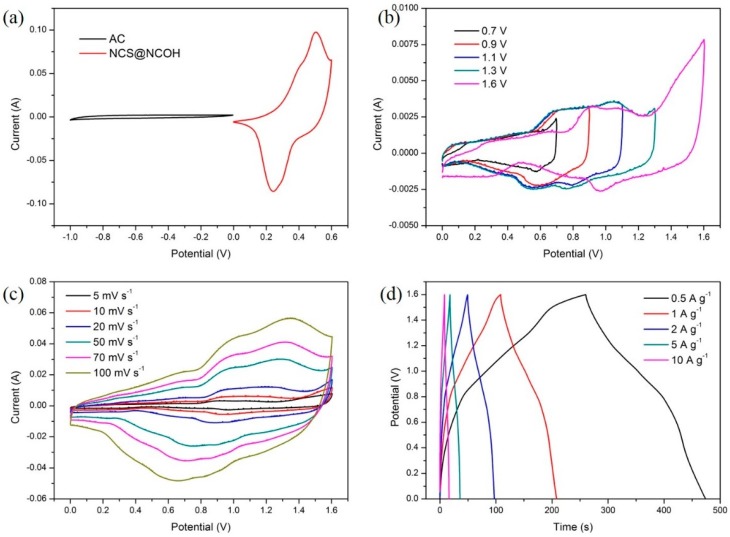
(**a**) CV curves of AC and NCS@NCOH at a scan rate of 5 mV s^−1^, (**b**) CV curves with different scan voltage windows, (**c**) CV curves of NCS@NCOH//AC at various scan rates, and (**d**) GCD curves of NCS@NCOH//AC at various current densities.

To examine the energy storage performance of the NCS@NCOH electrodes for practical applications, we built a (battery-type) device using AC as the negative electrode and an NCS@NCOH positive electrode in a 6 M KOH solution (as presented in Figure 10). Based on the charge balance principle of the electrodes, the mass ratios of AC to NCS@NCOH were controlled at approximately 2.3:1 in the ASCs, which conversely reveals the superior energy storage capability of NCS@NCOH deposited on the Ni foam. A comparison between the CV curves of the AC and NCS@NCOH at a scan rate of 5 mV in a conventional three-electrode system is illustrated in Fig. 11. As can be seen, the negative and positive electrodes are within a potential window of -1.0 to 0 V and 0 to 0.6 V, respectively. Therefore, the device should work at a voltage of 1.6 V. Figure 11b shows the CV curves of the device at a voltage window of 0.7–1.6 V and a scan rate of 5 mV s^−1^. Thus, the device with a stable electrochemical window of 0–1.6 V must be adjusted. The CV curves of the optimized NCS@NCOH//AC ASCs within the voltage window of 0–1.6 V at versatile scan rates are demonstrated in Figure 11. Figure 11 indicates that the ASCs achieve superior electric double-layer capacitive and pseudocapacitive performances owing to the quasi-rectangular shape with distinct peaks. In the CV curves, as the scan rate surges from 5 to 100 mV s^−1^, no clear distortion is identified, expressing the excellent capability rate of the ASCs and the quick charge-discharge reversibility. The GCD curves of the cell in diverse CDs are presented in Figure 11d. Following Equation (3), the areal capacities at CDs of 0.5, 1, 2, 5, and 10 Ag^−1^ are 66.7, 62.3, 59.4, 55.4, and 50.3 Fg^−1^, respectively. 

**Figure 12:**
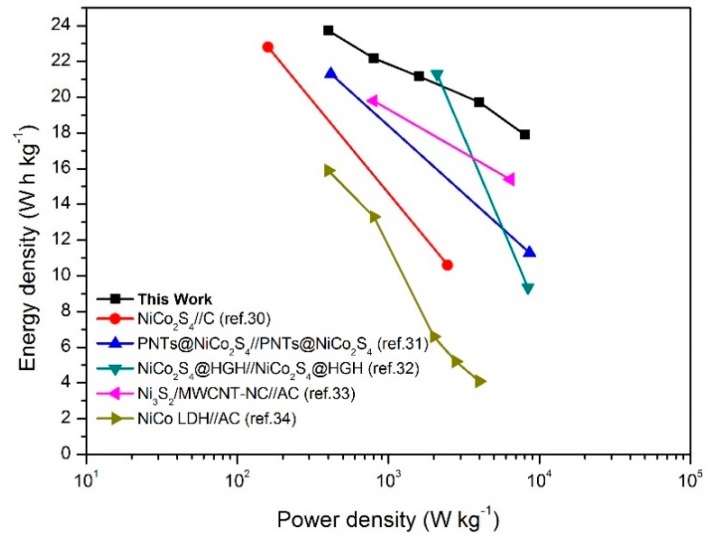
Ragone plots related to energy and power densities of NCS@NCOH//AC.

Figure 12 shows the Ragone plots of the NCS@NCOH//AC asymmetric supercapacitors attained from the GCD measurements at various CDs. Furthermore, following Equations (4) and (5), it can be seen that the NCS@NCOH//AC asymmetric supercapacitor achieves a maximum energy density of 23.7 Whkg^−1^ at a power density of 400 W kg^−1^ and can maintain an energy density of 17.9 Whkg^−1^ at a power density of 8 kWkg^−1^. When the current density is 0.5 Ag^−1^, the NiCo-LDH//AC asymmetric supercapacitor device exhibits a high-energy density of 15.9 Whkg^−1^ with a corresponding power density of 400 Wkg^−1^. As a result, the superior super-capacitive performance of the NCS@NCOH//AC device originates from the following characteristics: (1) The NCS nanosheets enhance the interfacial contact between the electrolyte and the active materials, and they provide abundant active sites for the energy storage properties; (2) the AC@NF negative electrode facilitates ion transport and electrolyte accessibility, leading to a lower resistance. From our experimental achievements, it can be stated that we obtained better results compared to the other reported nickel-cobalt sulfide-based ASCs such as NCS//C ASCs (22.8 Whkg^−1^ at 160 Wkg^−1^) [30], Co_3_O_4_@MnO_2_//MEGO (17.7 Whkg^−1^ at 158 Wkg^−1^), PNTs@NCS//PNTs@NCS ASCs (21.3 Whkg^−1^ at 417 Wkg^−1^) [31], NCS@HGH//NCS@HGH ASCs (21.3 Whkg^−1^ at 2100 Wkg^−1^) [32], Ni_3_S_2_/MWCNT-NC//AC ASCs (19.8 Whkg^−1^ at 798 Wkg^−1^) [33], and NiCo LDH//AC ASCs (15.9 Whkg^−1^ at 400 Wkg^−1^) [34]. 

## 4. Conclusions

We successfully synthesized a new non-binder electrode with a core-shell structure of NCS@NCOH on Ni foam, which was successfully devised and manufactured using a facile two-step hydrothermal path and subsequent electrodeposition. The outstanding electrochemical performance (energy storage) is attributed to a complex design using a highly conductive nickel-cobalt sulfide nanotube as the backbone of the NCS-LDH nanosheets, which can counteract the finite electric conductivity of the NC-LDH itself. For high-performance energy-storage devices, the NCS@NC-LDH nanostructure composite can be considered a potential electrode. The experimental findings indicate that the NCS@NCOH electrode achieves good cycle stability (retaining 65.1% at 50 Ag^−1^ for 3,000 cycles) and a high specific capacity (2105.1 Fg^−1^ at 2 Ag^−1^). Furthermore, a device (battery-type) was manufactured for practical application based on AC and NCS@NCOH as the negative and positive electrodes, supplying a high-energy density of 23.73 Whkg^−1^ at a power density of 400 Wkg^−1^ and a high rate stability. The experimental findings demonstrate that the synthesized electrode material fabricated using a hydrothermal technique followed by an electrochemical deposition method has a vast potential for application in energy-storage devices.
